# On the link between the topside ionospheric effective scale height and the plasma ambipolar diffusion, theory and preliminary results

**DOI:** 10.1038/s41598-020-73886-4

**Published:** 2020-10-16

**Authors:** Alessio Pignalberi, Michael Pezzopane, Bruno Nava, Pierdavide Coïsson

**Affiliations:** 1 Istituto Nazionale di Geofisica e Vulcanologia, Via di Vigna Murata 605, 00143 Rome, Italy; 2 The Abdus Salam International Centre for Theoretical Physics, Strada Costiera 11, 34151 Trieste, Italy; 3 Université de Paris, Institut de physique du globe de Paris, CNRS, F-75005 Paris, France

**Keywords:** Space physics, Atmospheric science, Space physics

## Abstract

Over the years, an amount of models relying on effective parameters were implemented in the challenging issue of the topside ionosphere description. These models are based on different analytical functions, but all of them depend on a parameter called effective scale height, that is deduced from topside electron density measurements. As their names state, they are effective in reproducing the topside electron density profile only when applied to the analytical function used to derive them. Then, in principle, they do not have any physical meaning. It is the goal of this paper to mathematically link the effective scale height modeled through the Epstein layer to the vertical scale height theoretically deduced from the plasma ambipolar diffusion theory. Firstly, effective and theoretical scale heights are linked through a mathematical relation by showing that they tend to each other in the topside ionosphere. Secondly, their connection is preliminarily demonstrated by calculating effective scale height values from the entire COSMIC/FORMOSAT-3 radio occultation dataset. Thirdly, a possible connection between the vertical gradient of the topside scale height (as obtained by COSMIC/FORMOSAT-3 satellites) and the electron temperature (as obtained by ESA Swarm B satellite) is studied by highlighting corresponding similarities in the diurnal, seasonal, solar activity, and latitudinal variability.

## Introduction

In the context of the Space Weather, the ionosphere plays a fundamental role as the medium coupling the external forcing given by solar radiation, solar wind, and magnetosphere, to the inner Earth’s atmosphere. Specifically, the topside part of the ionosphere, extending from the F-layer peak (corresponding to the ionospheric electron density maximum) to the plasmasphere, is of utmost importance because it contains the largest fraction of the ionospheric total electron content^1,2^.

In the topside, the electron density decreases monotonically as the ion population smoothly transitions from heavy O^+^ ions, dominating the lower part of the F region, to lighter H^+^ and He^+^ ions above. Moreover, the increase of the plasma temperature in the topside and the presence of the dynamical forcing imposed by magnetic and electric fields and by collisions with neutrals, makes the picture quite complex. The rate of electron density decrease in the topside is linked to several physical and chemical phenomena explained by the plasma ambipolar diffusion theory through the introduction of a theoretical vertical scale height^1–4^ (*VSH*). The *VSH* generalizes the concept of plasma scale height (*H*_p_) by including all the relevant physical and chemical concepts in its definition^3,4^. Specifically, *VSH* is equal to *H*_p_ only under diffusive equilibrium conditions, i.e., when the collisions with neutrals and the vertical gradient of plasma temperature can be neglected.

Despite their exact conceptual definition, both *VSH* and *H*_p_ are of difficult application for operational purposes. This is because they require a knowledge of the physical state of the plasma in terms of temperature, chemical state, and mean ion mass for the whole topside profile, that is currently unaffordable. This is why, in the past, a more direct and practical approach was developed based on the use of electron density measurements collected in the topside ionosphere by different techniques. Topside electron density measurements are limited both in space and time by the difficulties in probing an ionospheric region hidden to the widely spread ground-based ionosondes. More sophisticated and expensive techniques and instruments like topside sounders, radio occultation (RO) satellites, and incoherent scatter radars (ISR), were employed to this task. Over the years, several modeling techniques were developed to obtain the most reliable picture of the topside vertical electron density profile through an increasingly sophisticated description of the topside effective vertical scale height^5–19^ (*H*). These vertical scale heights gained the adjective “effective” because they are effective in reproducing the topside electron density profile only when applied to the analytical function used to derive them from either measured topside profiles or measured electron density values at low Earth orbit (LEO) satellite altitude. Different functions were applied by several authors, the most popular being exponential, Chapman, and Epstein families of functions^12,20–24^. The first studies were based on constant *H* values, but the growing bulk of topside measurements suggested the need to allow for an increasing trend of *H* with altitude^13,14,25–27^. This is why the most recent topside models are based on effective scale heights varying with altitude.

Despite the increasing complexity and reliability of modern topside models based on effective topside scale heights, in principle, modeled *H* values have no clear connection with theoretical *VSH*. This happens because these modeled *H* values do not rely on the physical quantities describing the topside electron density distribution (for example, ions distribution, plasma temperature, dynamics, and collisions), but only on the electron density profile itself which is indeed the final result of different physical mechanisms operating at the same time. This makes it difficult to associate the observed *H* variability to physical parameters, in order to perform a prediction of the topside ionosphere variability based on theoretical expectations^3,4,28^. The goal of this work is to shed some light on the connection between modeled *H* and theoretical *VSH* (as derived by plasma ambipolar diffusion theory) through mathematical deductions, modeling approaches, and measured topside datasets.

Recently, Pignalberi et al.^13^ have proposed a method to derive a fully analytical expression of the effective topside scale height based on the analytical inversion of the semi-Epstein function (applied by both International Reference Ionosphere^29^ (IRI) and NeQuick^30^ models to represent the topside profile) as a function of the electron density and F-layer peak parameters. They applied the proposed methodology to a selected reliable dataset of COSMIC/FORMOSAT-3 (hereafter, COSMIC for brevity) RO profiles by highlighting a very distinct linear increasing trend of *H* for the lowest part of the topside ionosphere. By linearly fitting calculated *H*, they were able to obtain the *H*’s intercept and slope values for each COSMIC topside profile. These coefficients allow to model *H*, hence the topside electron density profile, in the topside region probed by COSMIC satellites: from the height of the F-layer peak to about 800 km.

In this study, the connection between the fully analytical scale height *H* of Pignalberi et al.^13^ and the theoretical *VSH* (from plasma ambipolar diffusion theory) is mathematically studied. This is done to link empirical modeled effective parameters to theoretical physical quantities. After having analytically derived the relation between effective and theoretical parameters, preliminary applications are shown based on effective parameters derived from the entire COSMIC RO dataset (by applying the approach of Pignalberi et al.^13^) and measured parameters from the European Space Agency (ESA) Swarm mission.

## Linking the Epstein layer effective vertical scale height to the plasma ambipolar diffusion theory

### The plasma ambipolar diffusion theory for the topside ionosphere

The motion of plasma in the topside ionosphere is dominated by diffusion along magnetic field lines. For our purposes, we can consider the ionospheric plasma as a minor gas mixture, formed by electrons and ions, immersed in the major neutral atmosphere gas. When speaking about ions in the topside, we mean a mixture of O^+^, H^+^, and He^+^ ions, with relative percentage variable with altitude. Then, motions of ions and electrons can be described by the Navier–Stokes equation of motion for both charged plasma components. Here, to derive the vertical scale height, we follow the simplified description of plasma diffusion made by Rishbeth and Garriott^1^ and Ratcliffe^2^. Specifically, only the vertical diffusion (along the *z* axis) is here considered. However, in the ionosphere F region, the plasma diffuses along the geomagnetic field lines. The extension to the general case, i.e., plasma diffusion along sloped geomagnetic field lines, is straightforward by considering the field lines inclination^1,2^.

Equations of motion for ions and electrons in the vertical component are:

1 $$\left\{ {\begin{array}{*{20}l} {\frac{\partial }{\partial z}\left( {N_{{\text{i}}} {\text{k}}_{{\text{B}}} T_{{\text{i}}} } \right) = - N_{{\text{i}}} m_{{\text{i}}} g + N_{{\text{i}}} eE_{{\text{z}}} - N_{{\text{i}}} m_{{\text{i}}} \nu_{{{\text{in}}}} \left( {W_{{\text{i}}} - W_{{\text{n}}} } \right),} \hfill \\ {\underbrace {{\frac{\partial }{\partial z}\left( {N_{{\text{e}}} {\text{k}}_{{\text{B}}} T_{{\text{e}}} } \right)}}_{{{\text{Pressure}}\;{\text{gradient}}}} = \underbrace {{ - N_{{\text{e}}} m_{{\text{e}}} g}}_{{{\text{Gravity}}\;{\text{field}}}} - \underbrace {{N_{{\text{e}}} eE_{{\text{z}}} }}_{{{\text{Electric}}\;{\text{field}}}} - \underbrace {{N_{{\text{e}}} m_{{\text{e}}} \nu_{{{\text{en}}}} \left( {W_{{\text{e}}} - W_{{\text{n}}} } \right)}}_{{{\text{Collisions}}\;{\text{with}}\;{\text{neutrals}}}},} \hfill \\ \end{array} } \right.$$

where the following physical quantities are defined:

- *N*_i_ and *N*_e_ are the ions and electrons densities, respectively;
- k_B_ is the Boltzmann’s constant;
- *T*_i_ and *T*_e_ are the ions and electrons temperatures, respectively;
- *m*_i_ and *m*_e_ are the ions and electrons masses, respectively;
- *g* is the acceleration due to the gravity field;
- *e* is the electric charge unit;
- *E*_z_ is the vertical component of the electric field;
- $\nu_{{{\text{in}}}}$ and $\nu_{{{\text{en}}}}$ are the ions-neutrals and electrons-neutrals effective collisions frequencies, respectively; collisions between charged particles are negligible in the topside ionosphere;
- *W*_i_ and *W*_e_ are the vertical component of ions and electrons drift velocities, respectively;
- *W*_n_ is the vertical component of neutrals velocity.

As described by Eq. (1), ions and electrons are both subject to their own pressure gradient, force of gravity, electromagnetic force imposed by electric fields, and collisions with neutrals. Ions and electrons are treated as perfect gases whose partial pressures are $P_{{\text{i}}} = N_{{\text{i}}} {\text{k}}_{{\text{B}}} T_{{\text{i}}}$ and $P_{{\text{e}}} = N_{{\text{e}}} {\text{k}}_{{\text{B}}} T_{{\text{e}}}$, respectively. However, electrical forces between ions and electrons prevent the separation of charges ensuring that they both diffuse at the same speed along magnetic field lines; hence the name “ambipolar” plasma diffusion. As a consequence, neutrality of charges is assumed $N_{{\text{e}}} \simeq N_{{\text{i}}}$. Collisions with neutral particles tend to hamper plasma diffusion at a rate dependent on collisions frequencies and difference in charged-neutrals velocities. From Dalgarno^31^ and Dalgarno et al.^32^, $\nu_{{{\text{in}}}}$ and $\nu_{{{\text{en}}}}$ effective collisions frequencies depend primarily on neutrals density *N*_n_, secondly on neutrals mass *m*_n_ and temperature *T*_n_, and dominate in the lower ionosphere (E region). Moreover, vertical velocity due to neutral winds is important in the lower ionosphere but it can be neglected in the topside; so, $W_{{\text{n}}} \simeq 0$ is assumed in our calculations and $W_{i} \simeq W_{e} \equiv W_{D}$, where *W*_D_ is defined as the plasma drift velocity, due to ambipolar diffusion.

By making the aforementioned assumptions, Eq. (1) become:

2 $$\left\{ {\begin{array}{*{20}l} {\frac{\partial }{\partial z}\left( {N_{{\text{e}}} {\text{k}}_{{\text{B}}} T_{{\text{i}}} } \right) = - N_{{\text{e}}} m_{{\text{i}}} g + N_{{\text{e}}} eE_{{\text{z}}} - N_{{\text{e}}} m_{{\text{i}}} \nu_{{{\text{in}}}} W_{{\text{D}}} ,} \hfill \\ {\frac{\partial }{\partial z}\left( {N_{{\text{e}}} {\text{k}}_{{\text{B}}} T_{{\text{e}}} } \right) = - N_{{\text{e}}} m_{{\text{e}}} g - N_{{\text{e}}} eE_{{\text{z}}} - N_{{\text{e}}} m_{{\text{e}}} \nu_{{{\text{en}}}} W_{{\text{D}}} .} \hfill \\ \end{array} } \right.$$

In the topside, a mixture of O^+^ and lighter H^+^ and He^+^ ions composes the positive charged particles population; because negative ions are not present there, only electrons compose the negative charged particles population. Since $m_{{\text{e}}} \ll m_{{\text{i}}}$ then $m_{{\text{e}}} N_{{\text{e}}} \ll \, m_{{\text{i}}} N_{{\text{i}}}$. Moreover, in the topside ionosphere, it is very well verified that $m_{{\text{e}}} \nu_{{{\text{en}}}} \ll \, m_{{\text{i}}} \nu_{{{\text{in}}}}$. So, summing up Eq. (2) for ions and electrons, we get:

3 $$\frac{\partial }{\partial z}\left( {N_{{\text{e}}} {\text{k}}_{{\text{B}}} T_{{\text{p}}} } \right) = - N_{{\text{e}}} mg - N_{{\text{e}}} m\nu W_{{\text{D}}} ,$$

where $m_{{\text{i}}} + m_{{\text{e}}} \simeq m_{{\text{i}}} \equiv m$, $\nu_{{{\text{in}}}} \equiv \nu$, and the plasma temperature has been defined as $T_{{\text{p}}} \equiv T_{{\text{e}}} + T_{{\text{i}}}$.

By solving the vertical derivative in the left-hand side of Eq. (3) and by grouping all terms in *N*_e_, the following expression for the logarithmic derivative of the electron density is obtained:

4 $$- \frac{1}{{N_{{\text{e}}} }}\frac{{\partial N_{{\text{e}}} }}{\partial z} = \frac{mg}{{{\text{k}}_{{\text{B}}} T_{{\text{p}}} }} + \frac{m\nu }{{{\text{k}}_{{\text{B}}} T_{{\text{p}}} }}W_{{\text{D}}} + \frac{1}{{T_{{\text{p}}} }}\frac{{\partial T_{{\text{p}}} }}{\partial z}.$$

Equation (4) describes the electron density variation with height in the plasma ambipolar diffusion state, and defines two important plasma parameters:

- The plasma scale height $H_{{\text{p}}} \equiv \frac{{{\text{k}}_{{\text{B}}} T_{{\text{p}}} }}{mg}$, that is the scale height describing the plasma distribution with height if only pressure gradient and gravity force were present;
- The plasma diffusion coefficient $D \equiv \frac{{{\text{k}}_{{\text{B}}} T_{{\text{p}}} }}{m\nu }$, which describes the effects of collisions for a minor plasma diffusing in the major neutrals constituents.

Correspondingly, Eq. (4) can be written as:

5 $$- \frac{1}{{N_{{\text{e}}} }}\frac{{\partial N_{{\text{e}}} }}{\partial z} = \frac{1}{{H_{{\text{p}}} }} + \frac{{W_{{\text{D}}} }}{D} + \frac{1}{{T_{{\text{p}}} }}\frac{{\partial T_{{\text{p}}} }}{\partial z}.$$

The vertical scale height *VSH* is defined as:

6 $$VSH \equiv \left( { - \frac{1}{{N_{{\text{e}}} }}\frac{{\partial N_{{\text{e}}} }}{\partial z}} \right)^{ - 1} ,$$

and gives the rate of change of the electron density with height.

By equating Eqs. (5) and (6), and solving for *VSH*, we obtain:

7 $$\, VSH = \frac{{{\text{k}}_{{\text{B}}} T{}_{{\text{p}}}}}{{mg + m\nu W_{{\text{D}}} + {\text{k}}_{{\text{B}}} \frac{{\partial T_{{\text{p}}} }}{\partial z}}}.$$

Equation (7) is the vertical scale height of the topside electron density as deduced from plasma ambipolar diffusion theory. *VSH* represents the generalization of *H*_p_ considering also the collisions with neutrals and the vertical gradient of plasma temperature; so, including also the effects of the thermal structure and dynamics of the ionosphere. *VSH* is equal to *H*_p_ only under diffusive equilibrium conditions, i.e., when *W*_D_ = 0, and when the vertical gradient of the plasma temperature can be neglected. Titheridge^33^ studying the behavior of the topside ionosphere under diffusive equilibrium (then without considering drifts and collisions), came to the conclusion that three physical parameters are the most important: (1) ion composition, (2) plasma temperature, (3) vertical gradient of the plasma temperature. Equation (7) states also the importance of considering the collisions and plasma drift velocity for a complete description. Relationships between *VSH* and *H*_p_ for different conditions were studied by Liu et al.^3,4^ through Millstone Hill (288.5°E, 42.6°N) and Arecibo (18.3°N, 293.2°E) ISR measurements, and were linked to changes in the shape of the F2-layer region by Luan et al.^28^. They found that the ratio between *VSH* and *H*_p_ shows distinct diurnal, seasonal, solar activity, and geographic latitude variations; thus, making the inclusion of the thermal structure and dynamics of the ionosphere, in the estimation of the scale height, of utmost importance for a better description of the topside profile. Anyway, it has to be kept in mind that the diffusive equilibrium condition is an idealized picture of the real topside ionosphere describing its asymptotic behavior in a rather simple analytical way^34^. For a complete and exact description of the topside ionosphere, the full time-dependent continuity equations for electrons and ions should be considered.

### Deriving the effective vertical scale height from topside electron density profiles

To calculate topside effective scale height values, the semi-Epstein analytical formulation is used^13,21,23,30^. The semi-Epstein analytical formulation describes the topside electron density *N*_e_ as a function of the reduced height $z = h - hm{\text{F2}}$, starting from the F2-layer peak density *Nm*F2 value at *z* = 0 (*h* = *hm*F2):

8 $$N_{{\text{e}}} (z) = 4Nm{\text{F2}}\frac{{\exp \left( {\frac{z}{{H_{{{\text{Epstein}}}} \left( z \right)}}} \right)}}{{\left[ {1 + \exp \left( {\frac{z}{{H_{{{\text{Epstein}}}} \left( z \right)}}} \right)} \right]^{2} }}.$$

The rate of the electron density decrease in the topside is driven by the topside effective scale height *H*_Epstein_(*z*) as described in Pignalberi et al.^13^:

9 $$H_{{{\text{Epstein}}}} (z) = \frac{z}{{\ln \left\{ {\frac{1}{{N_{{\text{e}}} (z)}}\left[ {\left( {2Nm{\text{F2}} - N_{{\text{e}}} (z)} \right) + 2\sqrt {Nm{\text{F2}}^{2} - N_{{\text{e}}} (z) \cdot Nm{\text{F2}}} } \right]} \right\}}}.$$

**Figure 1:**
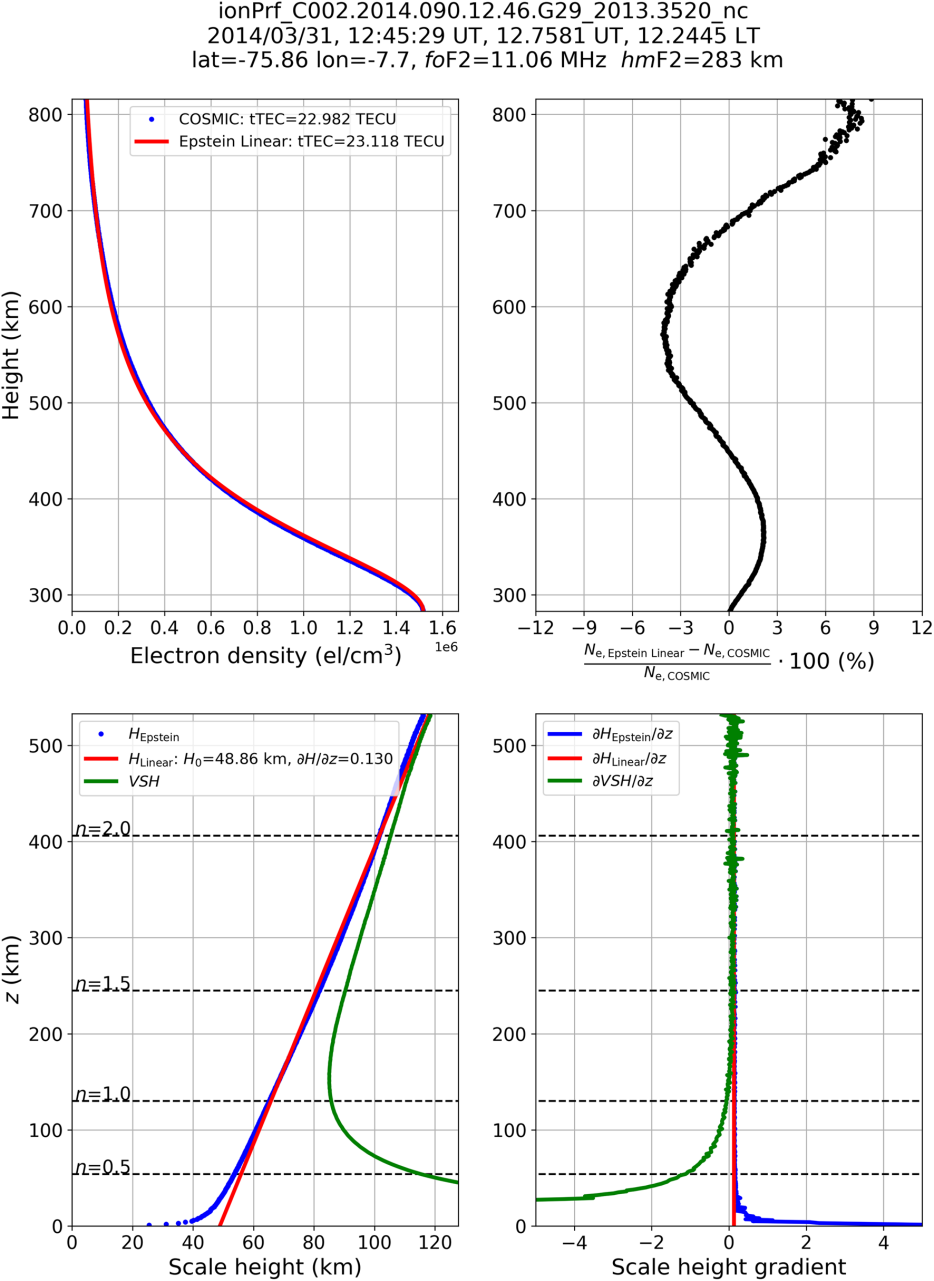
(Upper left panel) Topside electron density values retrieved by COSMIC (blue) and modeled by using the linear topside effective scale height *H*_Linear_(*z*) (red). (Upper right panel) Residuals percentage (black) between modeled (*N*_e, Epstein Linear_) and measured (*N*_e, COSMIC_) electron density values represented in the upper left panel. (Lower left panel) Topside effective scale height values (*H*_Epstein_(*z*), blue) obtained from the COSMIC measured profiles shown in the upper left panel, and corresponding modeling through a linear fit (*H*_Linear_(*z*), red). Green curves represent the theoretical *VSH* calculated from Eq. (13). (Lower right panel) Topside scale height gradient values for *H*_Epstein_(*z*) (blue), *H*_Linear_(*z*) (red), and *VSH* (green). Vertical coordinates are given as (top panels) height above Earth’s surface and (bottom panels) reduced height *z*. Horizontal dashed lines in the bottom panels represent different values of *n* = *z*/2*H*_Epstein_(*z*). In the title, the name of the considered ionPrf file along with time, spatial, and F2-layer peak parameters are reported. Specifically, the file ionPrf_C002.2014.090.12.46.G29_2013.3520_nc represents a high-latitude topside profile.

**Figure 2:**
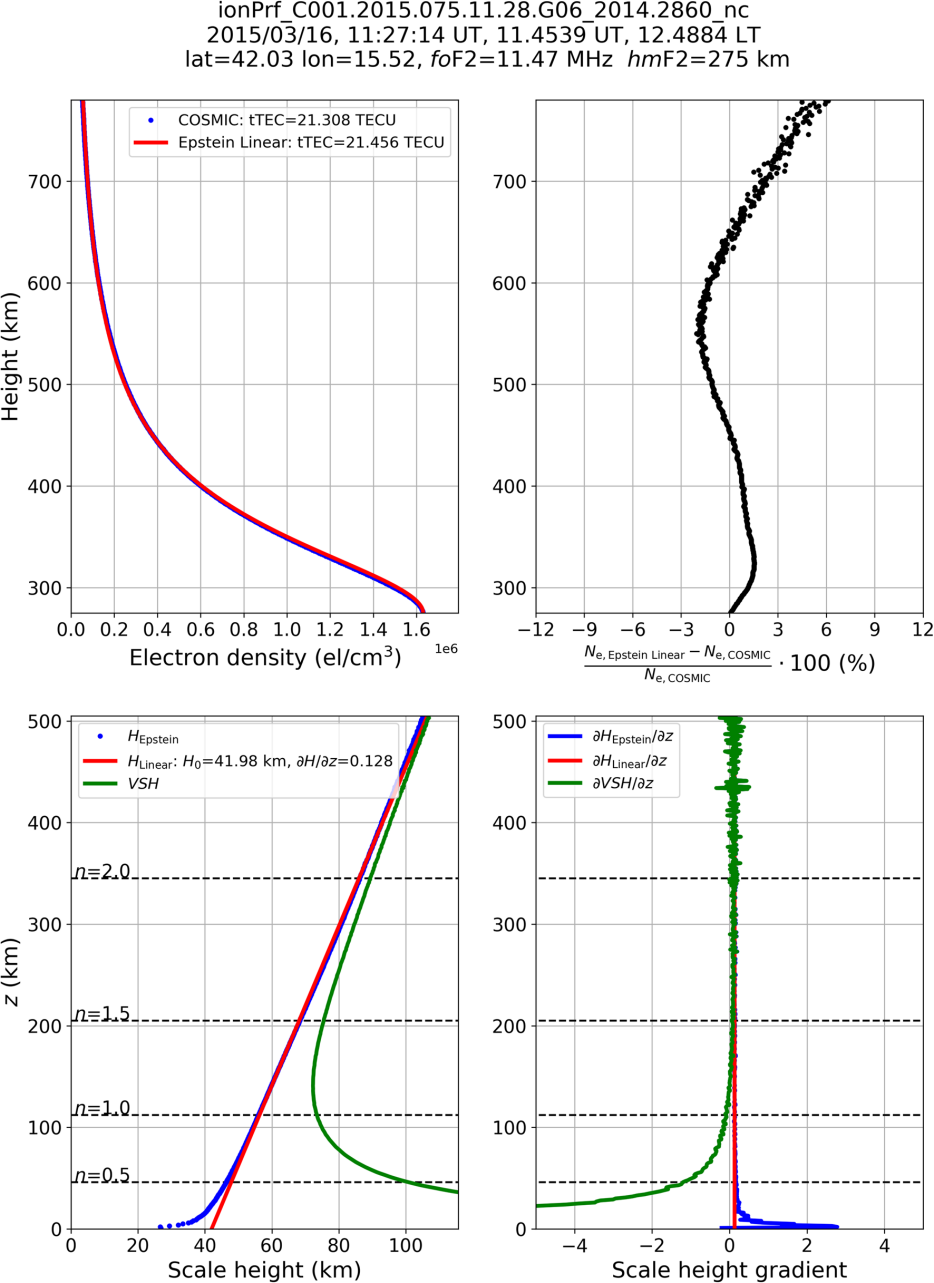
Same as Fig. 1 but for the file ionPrf_C001.2015.075.11.28.G06_2014.2860 representing a mid-latitude topside profile.

**Figure 3:**
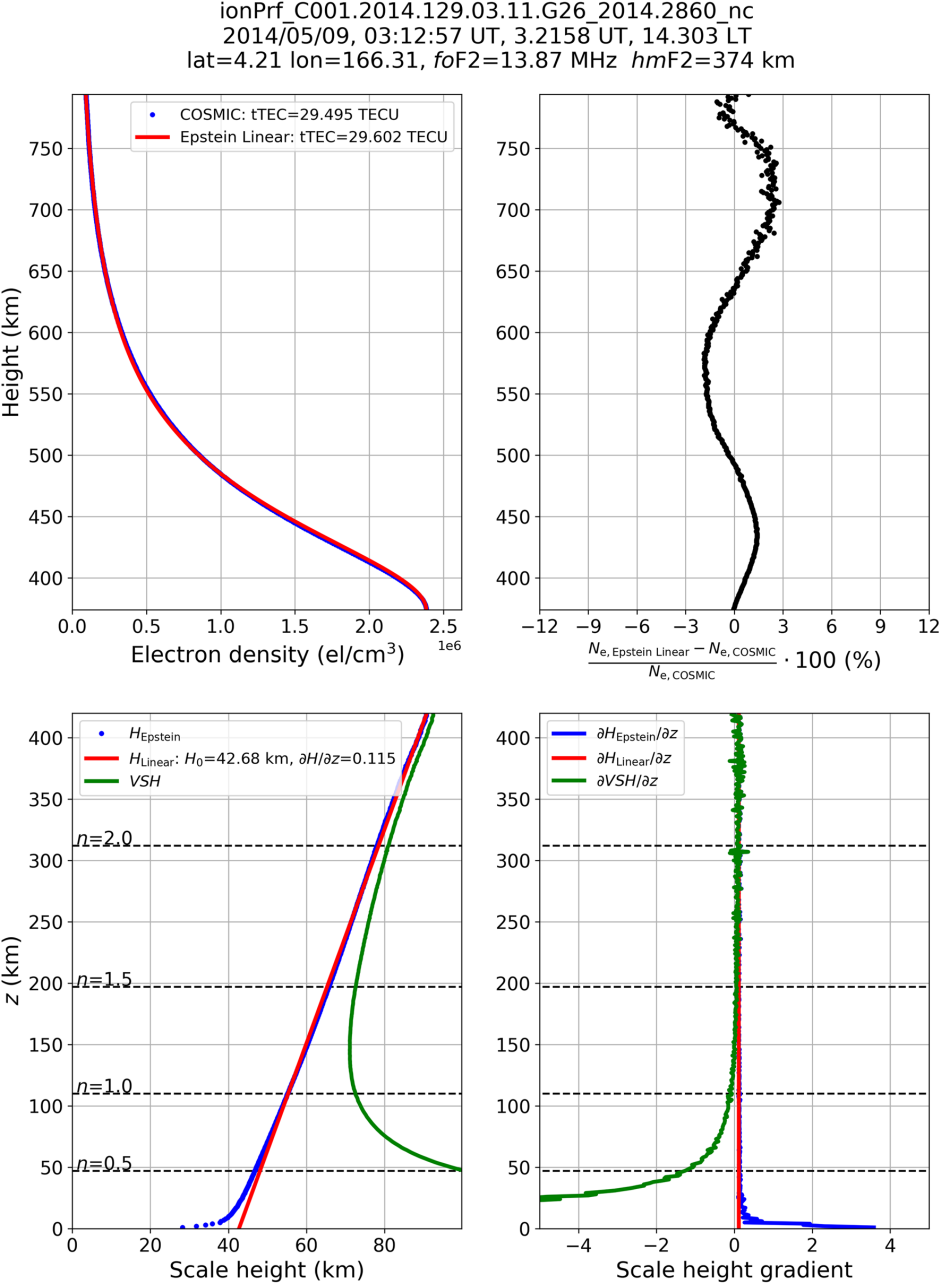
Same as Fig. 1 but for the file ionPrf_C001.2014.129.03.11.G26_2014.2860_nc representing a low-latitude topside profile.

By applying Eq. (9), it was possible to calculate the topside effective scale height *H*_Epstein_(*z*) for each height *z* by using COSMIC retrieved *N*_e_(*z*), *Nm*F2, and *hm*F2 values^13^. Some examples of this procedure are shown in Figs. 1, 2, and 3 for three selected COSMIC retrieved RO profiles. Blue points in the lower left panels of Figs. 1, 2, and 3 are *H*_Epstein_(*z*) values calculated by applying Eq. (9) to COSMIC retrieved electron density values, shown as blue points in the upper left panels of Figs. 1, 2, and 3. For a detailed description of the methodology refer to Pignalberi et al.^13^.

The logarithmic derivative of the electron density for a semi-Epstein layer can be analytically deduced by differentiating Eq. (8) with respect to the reduced height:

10 $$\frac{{\partial N_{{\text{e}}} (z)}}{\partial z} = \frac{{4Nm{\text{F2}}}}{{H_{{{\text{Epstein}}}} \left( z \right)}}\frac{{\exp \left( {\frac{z}{{H_{{{\text{Epstein}}}} \left( z \right)}}} \right)}}{{\left[ {1 + \exp \left( {\frac{z}{{H_{{{\text{Epstein}}}} \left( z \right)}}} \right)} \right]^{2} }}\left[ {\frac{{1 - \exp \left( {\frac{z}{{H_{{{\text{Epstein}}}} \left( z \right)}}} \right)}}{{1 + \exp \left( {\frac{z}{{H_{{{\text{Epstein}}}} \left( z \right)}}} \right)}}} \right].$$

The right-hand side of Eq. (10) can be simplified by considering Eq. (8) and by using the definition and properties of the hyperbolic tangent:

11 $$\frac{{\partial N_{{\text{e}}} (z)}}{\partial z} = - \frac{{N_{{\text{e}}} (z)}}{{H_{{{\text{Epstein}}}} \left( z \right)}}\tanh \left( {\frac{z}{{2H_{{{\text{Epstein}}}} \left( z \right)}}} \right).$$

Rearranging Eq. (11) to explicit the logarithmic derivative of the electron density, as done for Eq. (5), we obtain:

12 $$- \frac{1}{{N_{{\text{e}}} (z)}}\frac{{\partial N_{{\text{e}}} (z)}}{\partial z} = \frac{{\tanh \left( {\frac{z}{{2H_{{{\text{Epstein}}}} \left( z \right)}}} \right)}}{{H_{{{\text{Epstein}}}} \left( z \right)}}.$$

The reciprocal of Eq. (12)

13 $$VSH = \frac{{H_{{{\text{Epstein}}}} \left( z \right)}}{{\tanh \left( {\frac{z}{{2H_{{{\text{Epstein}}}} \left( z \right)}}} \right)}}.$$

describes the vertical scale height (Eq. (6)) for a topside electron density profile modeled through a semi-Epstein function (Eq. (8)) with a height-dependent effective scale height (Eq. (9)).

### Linking the theory to retrieved effective parameters

Equation (13) links the effective scale height parameter *H*_Epstein_(*z*) to the theoretical *VSH*, which depends on physical variables as described by Eq. (7). It is worth noting that, by its nature, *VSH* is always dependent on *z*, even where not explicitly written. Retrieving information on the effective scale height *H*_Epstein_(*z*) is much simpler than getting information on plasma temperature, ions distribution, and other chemical and dynamical quantities needed to calculate *VSH*, because only the knowledge of the topside electron density profile is required. Then, through the calculation of *H*_Epstein_(*z*) and Eq. (13), it is possible to infer information about *VSH* and then on the physical state of the topside ionosphere, under the simplified hypotheses imposed by the plasma ambipolar diffusion theory together with the imposition of Epstein layers.

In the section “A preliminary application based on COSMIC/FORMOSAT-3 Radio Occultation and ESA Swarm satellites data”, a preliminary application of Eq. (13) based on COSMIC RO topside profiles will be given; however, it is here interesting to deduce some general mathematical properties of *VSH* and *H*_Epstein_(*z*). First, the behavior of the ratio between *H*_Epstein_(*z*) and *VSH* at infinity is studied:

14 $$\mathop {\lim }\limits_{z \to \infty } \frac{{H_{{{\text{Epstein}}}} \left( z \right)}}{VSH} = \mathop {\lim }\limits_{z \to \infty } \tanh \left( {\frac{z}{{2H_{{{\text{Epstein}}}} \left( z \right)}}} \right) = 1 \Rightarrow H_{{{\text{Epstein}}}} \left( z \right)\xrightarrow[z \to \infty]{} VSH.$$

Equation (14) shows that *H*_Epstein_(*z*) tends to *VSH* at infinity but, it is interesting to highlight how rapidly this happens; then, a relation between the vertical derivative of *VSH* and *H*_Epstein_(*z*) has to be found. This is why, Eq. (13) is differentiated with respect to the reduced height:

15 $$\frac{\partial VSH}{{\partial z}} = \frac{{\frac{{\partial H_{{{\text{Epstein}}}} \left( z \right)}}{\partial z}}}{{\tanh \left( {\frac{z}{{2H_{{{\text{Epstein}}}} \left( z \right)}}} \right)}} - \frac{{H_{{{\text{Epstein}}}} \left( z \right) - z\frac{{\partial H_{{{\text{Epstein}}}} \left( z \right)}}{\partial z}}}{{2H_{{{\text{Epstein}}}} \left( z \right)\sinh^{2} \left( {\frac{z}{{2H_{{{\text{Epstein}}}} \left( z \right)}}} \right)}}.$$

Also, in this case the corresponding behavior at infinity is studied:

16 $$\mathop {\lim }\limits_{z \to \infty } \frac{\partial VSH}{{\partial z}} = \mathop {\lim }\limits_{z \to \infty } \frac{{\frac{{\partial H_{{{\text{Epstein}}}} \left( z \right)}}{\partial z}}}{{\tanh \left( {\frac{z}{{2H_{{{\text{Epstein}}}} \left( z \right)}}} \right)}} - \frac{{H_{{{\text{Epstein}}}} \left( z \right) - z\frac{{\partial H_{{{\text{Epstein}}}} \left( z \right)}}{\partial z}}}{{2H_{{{\text{Epstein}}}} \left( z \right)\sinh^{2} \left( {\frac{z}{{2H_{{{\text{Epstein}}}} \left( z \right)}}} \right)}} = \frac{{\partial H_{{{\text{Epstein}}}} \left( z \right)}}{\partial z}.$$

Equation (16) shows that $\frac{\partial VSH}{{\partial z}}$ tends to $\frac{{\partial H_{{{\text{Epstein}}}} \left( z \right)}}{\partial z}$ at infinity, then both the values and the derivatives with height of *H*_Epstein_(*z*) and *VSH* tend to be equal very distant from the F2-layer peak (virtually at infinity).

To be valuable for operational purposes, it is desirable that relations (14) and (16) are valid (within few percent of error) also in the lower topside ionosphere, i.e., in the region between the F2-layer peak (highly variable between around 200 and 400 km) and the upper transition height (the height that separates the ionosphere from the plasmasphere, highly variable in a wide range of altitudes around 1000 km). In order to verify this desired behavior, we further developed the calculation, with the change of variable $z = n \cdot 2H_{{{\text{Epstein}}}} \left( z \right)$, $n \in {\mathbb{R}}_{0}^{ + }$, and evaluated *VSH* and corresponding derivatives through Eqs. (13) and (15):

17 $$\left\{ {\begin{array}{*{20}l} {\left. {VSH} \right|_{{z = n \cdot 2H_{{{\text{Epstein}}}} \left( z \right)}} = \frac{{H_{{{\text{Epstein}}}} \left( z \right)}}{\tanh \left( n \right)},} \hfill \\ {\left. {\frac{\partial VSH}{{\partial z}}} \right|_{{z = n \cdot 2H_{{{\text{Epstein}}}} \left( z \right)}} = \frac{{\partial H_{{{\text{Epstein}}}} \left( z \right)}}{\partial z}\left[ {\frac{{n\tanh \left( n \right) + \sinh^{2} \left( n \right)}}{{\tanh \left( n \right)\sinh^{2} \left( n \right)}}} \right] - \frac{1}{{2\sinh^{2} \left( n \right)}}.} \hfill \\ \end{array} } \right.$$

Numerical values of *VSH* and $\frac{\partial VSH}{{\partial z}}$ for increasing *n* values (then for increasing heights, where now the height is given as multiples of *H*_Epstein_(*z*)), with corresponding numerical values of coefficients of Eq. (17), are given in Table 1. Table 1 clearly shows how *VSH* and $\frac{\partial VSH}{{\partial z}}$ tend very quickly to *H*_Epstein_(*z*) and $\frac{{\partial H_{{{\text{Epstein}}}} \left( z \right)}}{\partial z}$, respectively. Already for *n* = 2, i.e., for *z* = 4 *H*_Epstein_(*z*), *VSH* and *H*_Epstein_(*z*) are the same within 4% and are virtually the same for *n* = 3. For *n* = 0, i.e., at the F2-layer peak, *VSH* and *H*_Epstein_ are not comparable because of the mathematical definition of the semi-Epstein layer and the corresponding effective scale height, which is indefinite at *z* = 0. However, this is only a mathematical issue which does not cause any problem for operational applications. In fact, for the *N*_e_ calculation (Eq. (8)) what is important is the ratio between *z* and *H*_Epstein_, and this ratio behaves well for $z \to 0$ (as showed by Pignalberi et al.^13^). A demonstration of the mathematical results shown by Table 1 is given in the section “Deriving the effective vertical scale height from Radio Occultation data” based on retrieved COSMIC RO topside electron density profiles.

**Table 1:** Numerical values of *VSH* and ${{\partial VSH} \mathord{\left/ {\vphantom {{\partial VSH} {\partial z}}} \right. \kern-\nulldelimiterspace} {\partial z}}$ as a function of *H*_Epstein_(*z*) and ${{\partial H_{{{\text{Epstein}}}} \left( z \right)} \mathord{\left/ {\vphantom {{\partial H_{{{\text{Epstein}}}} \left( z \right)} {\partial z}}} \right. \kern-\nulldelimiterspace} {\partial z}}$, respectively, and related numerical coefficients defined in Eq. (17), for different values of *n* = *z*/2*H*_Epstein_(*z*).

$n$	$\tanh \left( n \right)$	$\left. {VSH} \right|_{{z = n \cdot 2H_{{{\text{Epstein}}}} \left( z \right)}}$	$\frac{{n\tanh \left( n \right) + \sinh^{2} \left( n \right)}}{{\tanh \left( n \right)\sinh^{2} \left( n \right)}}$	$\frac{1}{{2\sinh^{2} \left( n \right)}}$	$\left. {\frac{\partial VSH}{{\partial z}}} \right|_{{z = n \cdot 2H_{{{\text{Epstein}}}} \left( z \right)}}$
0	0	∞	∞	∞	∞
1	0.762	$1.313 \cdot H_{{{\text{Epstein}}}} \left( z \right)$	2.037	0.362	$2.037 \cdot \displaystyle \frac{{\partial H_{{{\text{Epstein}}}} \left( z \right)}}{\partial z} - 0.362$
2	0.964	$1.037 \cdot H_{{{\text{Epstein}}}} \left( z \right)$	1.189	0.038	$1.189 \cdot \displaystyle \frac{{\partial H_{{{\text{Epstein}}}} \left( z \right)}}{\partial z} - 0.038$
3	0.995	$1.005 \cdot H_{{{\text{Epstein}}}} \left( z \right)$	1.035	0.005	$1.035 \cdot \displaystyle \frac{{\partial H_{{{\text{Epstein}}}} \left( z \right)}}{\partial z} - 0.005$
4	0.999	$1.001 \cdot H_{{{\text{Epstein}}}} \left( z \right)$	1.006	0.001	$1.006 \cdot \displaystyle \frac{{\partial H_{{{\text{Epstein}}}} \left( z \right)}}{\partial z} - 0.001$

## A preliminary application based on COSMIC/FORMOSAT-3 radio occultation and ESA Swarm satellites data

The mathematical findings obtained in the section “Linking the Epstein layer effective vertical scale height to the plasma ambipolar diffusion theory” are here substantiated by applying them to actual measured data. For this purpose, the very reliable and wide dataset of topside electron density vertical profiles given by COSMIC RO is exploited. The Pignalberi et al.^13^ approach is first applied to the entire COSMIC dataset to retrieve effective scale height parameters on a global basis and for different helio-geophysical conditions. After that, a preliminary comparison between these retrieved effective scale height parameters and physical quantities measured by ESA’s Swarm satellites is given.

### Deriving the effective vertical scale height from radio occultation data

Pignalberi et al.^13^ performed a study based on a selected dataset of 382 COSMIC/FORMOSAT-3 RO profiles matching simultaneously measured F2-layer peak parameters (*Nm*F2 and *hm*F2) by co-located ionosondes. This allowed to work on a very reliable dataset of topside electron density profiles^35^. The Pignalberi et al.^13^ technique is here applied to the entire COSMIC RO dataset, recorded from 2006 to 2018, to retrieve preliminary information on the effective vertical scale height dependence on diurnal, seasonal, solar activity, and spatial variabilities.

COSMIC/FORMOSAT-3 was a six microsatellites constellation launched on 15 April 2006 and deployed into a circular orbit (with 72° of inclination) at about 800 km of altitude (reached at the end of 2007) and a separation angle of 30° in longitude between neighboring satellites^36^. The mission was a collaborative project between the National Space Organization in Taiwan and the University Corporation for Atmospheric Research in the United States. Each satellite carried a Global Positioning System (GPS) RO receiver, composed by four antennas, capable of measuring the phase delay of radio waves from GPS satellites as they are occulted by the Earth’s atmosphere, thus providing an accurate determination of the ionospheric vertical electron density profile. COSMIC RO data (*ionPrf* files) were downloaded from the COSMIC Data Analysis and Archive Center (CDAAC, https://cdaac-www.cosmic.ucar.edu/cdaac/products.html).

In this work, all available COSMIC ionPrf files from 22 April 2006 to 31 December 2018 were used, a total of 3,626,729 COSMIC retrieved electron density profiles. For the selection of reliable COSMIC topside profiles, a specific filtering process was developed to remove profiles affected by issues related to the failure of the spherical symmetry assumption in the Abel inversion procedure^37^. The interested reader can refer to the section “Methods” for a detailed description of the filtering process. After filtering, the COSMIC dataset used in this study reduced to 1,791,676 topside profiles (49.4% of the total).

In Pignalberi et al.^13^, *H*_Epstein_(*z*) values retrieved by COSMIC RO topside electron density values through Eq. (9) were linearly fitted to calculate corresponding slope and intercept values. The topside effective scale height thus obtained, called *H*_Linear_(*z*), is function of the reduced height *z*:

18 $$\left\{ \begin{gathered} H_{{{\text{Linear}}}} (z) = \frac{{\partial H_{{{\text{Linear}}}} }}{\partial z}z + H_{{0,{\text{ Linear}}}} , \hfill \\ H_{{0,{\text{ Linear}}}} = H_{{{\text{Linear}}}} (z = 0) = H_{{{\text{Linear}}}} (h = hm{\text{F2}}). \hfill \\ \end{gathered} \right.$$

In Eq. (18), ${{\partial H_{{{\text{Linear}}}} } \mathord{\left/ {\vphantom {{\partial H_{{{\text{Linear}}}} } {\partial z}}} \right. \kern-\nulldelimiterspace} {\partial z}}$ and *H*_0, Linear_ are the slope and intercept values obtained after applying the linear fit procedure of Pignalberi et al.^13^. The slope represents the gradient of the modeled topside scale height ${{\partial H_{{{\text{Linear}}}} } \mathord{\left/ {\vphantom {{\partial H_{{{\text{Linear}}}} } {\partial z}}} \right. \kern-\nulldelimiterspace} {\partial z}}$, while the intercept *H*_0, Linear_ represents the value of *H*_Linear_(*z*) at the F2-layer peak (*h* = *hm*F2).

This procedure is here applied to each of the 1,791,676 selected COSMIC topside profiles, thus allowing for compiling a very large dataset of ${{\partial H_{{{\text{Linear}}}} } \mathord{\left/ {\vphantom {{\partial H_{{{\text{Linear}}}} } {\partial z}}} \right. \kern-\nulldelimiterspace} {\partial z}}$ and *H*_0,Linear_. Some examples of application of Eq. (18) are shown in Figs. 1, 2, and 3 for three COSMIC RO profiles recorded at high (Fig. 1), mid (Fig. 2), and low (Fig. 3) latitudes. In both examples, *H*_Linear_(*z*) values are obtained applying the linear fit of Eq. (18) to *H*_Epstein_(*z*) values obtained through Eq. (9). Modeled topside electron density values are thus calculated replacing *H*_Epstein_(*z*) with *H*_Linear_ in Eq. (8), and are represented by the red curve in the upper left panels of Figs. 1, 2, and 3.

Figures 1, 2, and 3 show how *H*_Epstein_(*z*) values retrieved from COSMIC *N*_e_ measurements exhibit a clear linear trend with height; while only a slight departure from this behavior is visible for the few first tens of kilometers above the F2-layer peak. As a consequence, the linear fitting procedure represented by Eq. (18) allows to reliably describe the topside effective scale height and to properly reproduce the topside electron density profile. Green curves in the lower left panels represent *VSH* values calculated through Eq. (13). As it was already mathematically demonstrated in the section “Linking the theory to retrieved effective parameters” and illustrated by Table 1 values, *VSH* markedly departs from *H*_Epstein_(*z*) just above the F2-layer peak (for *z* → 0 or *n* → 0) but rapidly approaches it at higher altitudes by assuming quite similar values above *n* > 2. In the lower right panels, the corresponding behavior of vertical gradients for *H*_Epstein_(*z*), *H*_Linear_(*z*), and *VSH*, are shown. Also in this case, theoretical *VSH* values depart from effective scale height values just above the F2-layer peak, but then rapidly tend to the effective values above.

Figures 1, 2, and 3 testify that it is possible to relate in a very accurate way effective scale height values to theoretical *VSH* ones for a wide range of altitudes from some hundreds of kilometers above the F2-layer peak to the plasmasphere domain.

The comparison made by Pignalberi et al.^13^ between electron density values measured by COSMIC and those modeled by using *H*_Linear_(*z*) showed that it is possible to accurately reproduce the ionospheric topside electron density profile by using a semi-Epstein layer with a topside scale height linearly dependent on the height, at least from *hm*F2 to about 800 km, considering a limited dataset of only 382 RO profiles.

For each of the 1,791,676 selected COSMIC topside profiles, the topside total electron content (tTEC) is calculated by integrating the topside electron density values from *hm*F2 to the satellite height. tTEC values are calculated for measured and modeled topside profiles:

19 $$\left\{ {\begin{array}{*{20}l} {{\text{tTEC}}_{{{\text{measured}}}} = \int\limits_{{hm{\text{F2}}}}^{{h_{{{\text{COSMIC}}}} }} {N_{{{\text{e}},{\text{COSMIC}}}} {\text{d}}h,} } \hfill \\ {{\text{tTEC}}_{{{\text{modeled}}}} = \int\limits_{{hm{\text{F2}}}}^{{h_{{{\text{COSMIC}}}} }} {N_{{\text{e,Epstein Linear}}} {\text{d}}h,} } \hfill \\ \end{array} } \right.$$

where *N*_e,COSMIC_ are electron density values measured by COSMIC, while *N*_e,Epstein Linear_ are those modeled by Eq. (8) with topside effective scale height values from Eq. (18).

To perform a statistical validation, tTEC Root Mean Square Error (RMSE) and Normalized Root Mean Square Error (NRMSE) values are calculated, expressed in TECU (1 TECU = 10^16^ el/m^2^) and in percentage, for the entire COSMIC dataset

20 $$\left\{ {\begin{array}{*{20}l} {{\text{RMSE [TECU]}} = \sqrt {\frac{{\mathop \sum \nolimits_{i = 1}^{N} \left( {{\text{tTEC}}_{{{\text{modeled,}}i}} - {\text{tTEC}}_{{{\text{measured,}}i}} } \right)^{2} }}{N},} } \hfill \\ {{\text{NRMSE [\% ]}} = \sqrt {\frac{{\mathop \sum \nolimits_{i = 1}^{N} \left( {\frac{{{\text{tTEC}}_{{{\text{modeled,}}i}} - {\text{tTEC}}_{{{\text{measured,}}i}} }}{{{\text{tTEC}}_{{{\text{measured,}}i}} }} \cdot 100} \right)^{2} }}{N}} ,} \hfill \\ \end{array} } \right.$$

where *N* is 1,791,676, the total number of selected COSMIC profiles.

**Figure 4:**
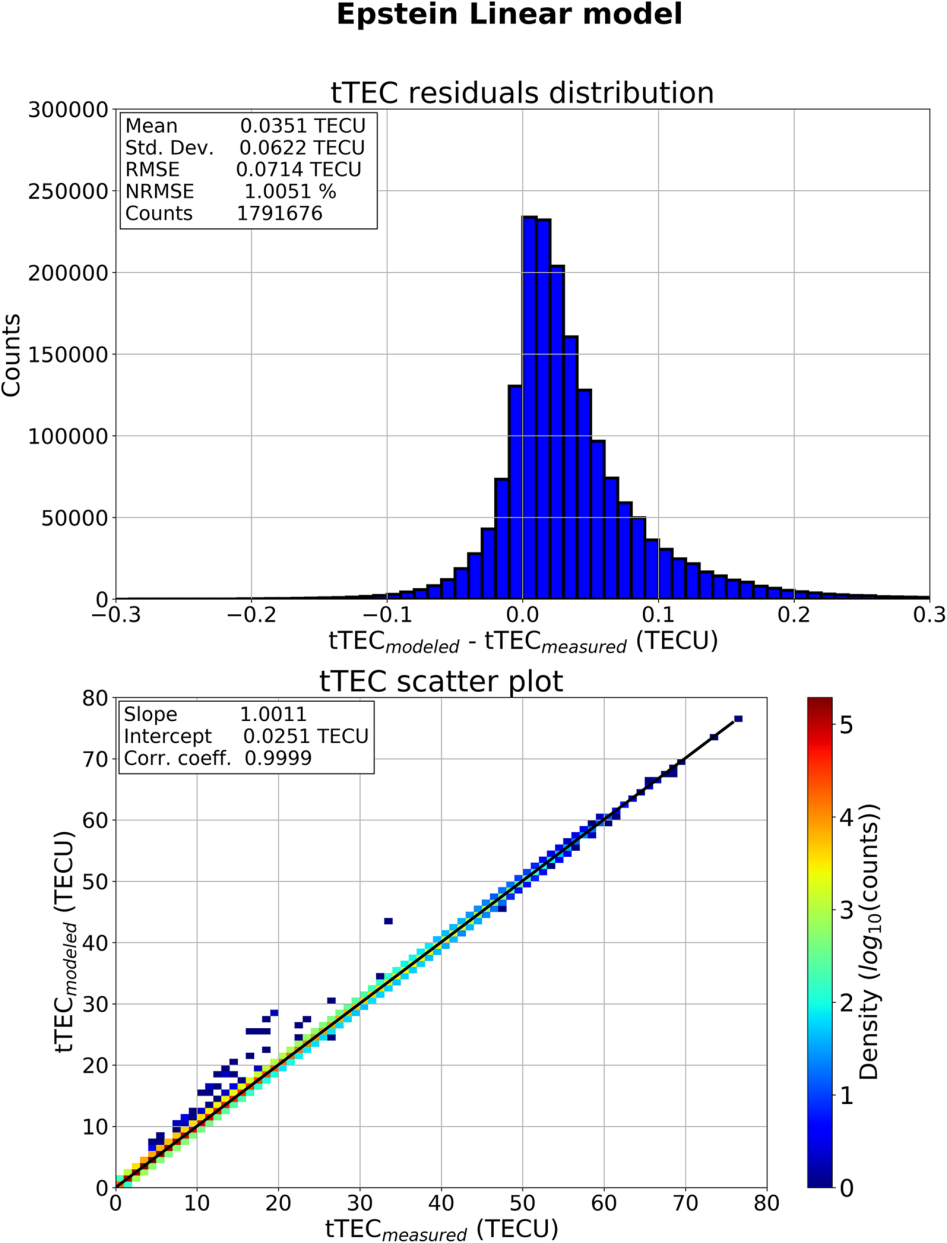
(Top panel) Histogram of residuals between modeled and measured tTEC values. (Bottom panel) Density plot of modeled and measured tTEC values. Black line represents the best linear fit line. The measured tTEC values are related to COSMIC RO profiles, while the modeled ones are obtained by applying the Epstein Linear formulation.

Top panel of Fig. 4 shows the histogram of residuals between modeled and measured tTEC values, with the corresponding statistical values: RMSE = 0.0714 TECU, NRMSE = 1.0051%. Moreover, the distribution is well peaked around zero (residuals mean = 0.0351 TECU) with a very low dispersion (residuals standard deviation = 0.0622 TECU). Bottom panel of Fig. 4 shows the scatter plot of modeled versus measured tTEC values. Scattered tTEC values are binned on a 1 TECU × 1 TECU grid, and the number of points in each bin is shown in color-coded logarithmic scale. The best linear fit is drawn as a solid black line. The scatter plot exhibits a clear one-to-one dependence between measured and modeled tTEC values (slope = 1.0011, intercept = 0.0251 TECU, Pearson correlation coefficient = 0.9999). These results testify that the application of Pignalberi et al.^13^ methodology on COSMIC RO topside profiles is very effective in modeling the topside effective vertical scale height through a linear fit procedure. Moreover, this is a further verification that the vertical scale height exhibits a very clear linear trend, at least for the altitudes probed by COSMIC satellites, from *hm*F2 to about 800 km.

Looking more closely at the residuals of Fig. 4, we can see that modeled values show a slight tendency to overestimate measured values. This is mainly due to the fact that the linear fit of calculated *H*_Epstein_(*z*) values is made starting from *hm*F2 + 50 km upwards^13^, thus producing a slight overestimation of modeled scale height values just above *hm*F2, which in turns leads to an overestimation of measured *N*_e_ values. On the contrary, tTEC values outside the linear fit in the scatter plot of Fig. 4 (the dark blue bins just above the fit line ) cannot be explained as above. These profiles do not exhibit a linear trend of the scale height with altitude. They can be considered as outliers and they are not visible in the histogram of Fig. 4 because outside the displayed axis range. Anyhow, these outliers affect very little the full statistics because they are relatively few (tens of topside profiles).

**Figure 5:**
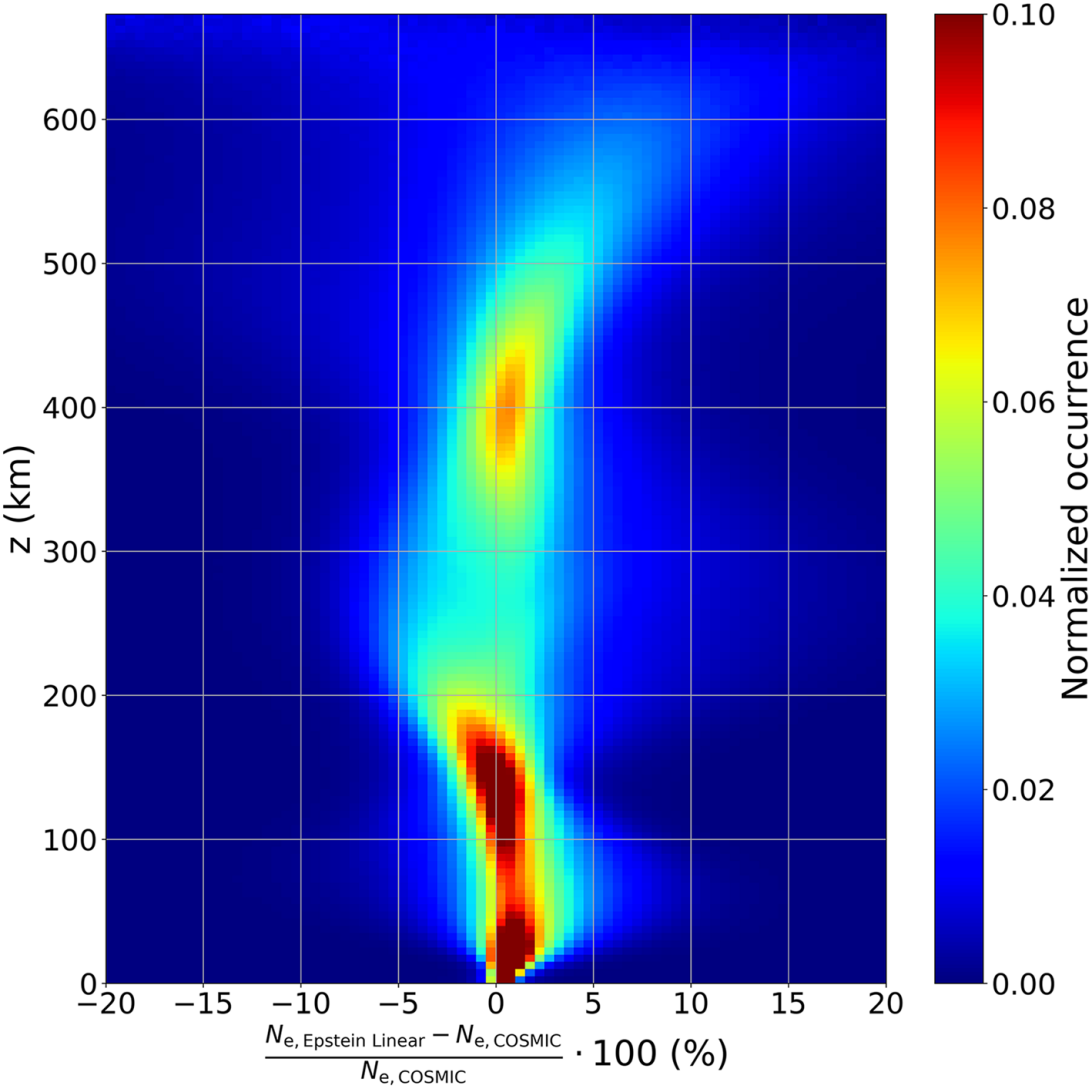
Density plot of residuals percentage between modeled (*N*_e, Epstein Linear_) and measured (*N*_e, COSMIC_) electron density values as a function of the reduced height *z*. The number of occurrences in each bin is normalized with respect to the number of occurrences for the same altitude.

Figure 5 shows the normalized occurrence of residuals percentage between modeled and measured electron density values, for different reduced heights, for the entire COSMIC selected dataset. Notably, it is expected that the spreading of data increases with altitude, because all profiles are constrained at the peak (*z* = 0). Nevertheless, up to *z* = 500 km the profiles are remarkably well reproduced. Specifically, Fig. 5 highlights that most of the occurrences lie in a narrow range between ± 5%. As a consequence, topside electron density values modeled by the *H*_Linear_(*z*) approximation (Eq. (18)) allow for a reliable description of the topside profile in the altitude range probed by COSMIC satellites.

Results of Figs. 4 and 5 confirm what preliminarily found by Pignalberi et al.^13^, i.e., the scale height retrieved from COSMIC RO profiles exhibits a clear linear trend for the lower topside region, the one from the F2-layer peak to about 800 km of height. The COSMIC RO dataset used for this work includes different diurnal, seasonal, and solar activity conditions spanning from equatorial to auroral latitudes. Then, the linear trend of the topside scale height is a very well defined topside ionospheric feature, regardless of geophysical conditions. It is worth noting that these results are valid until about 800 km of height (the maximum height covered by COSMIC satellites); for higher altitudes it has been demonstrated that a departure from the linearity takes place^14^.

### Comparison with ESA Swarm data

From Eq. (7), it is clear that several physical quantities account for the *VSH* variability. Specifically, plasma temperatures and corresponding vertical gradient values, ions distribution, ions-neutrals collision frequency, and plasma vertical drift velocity. Moreover, the variation with height of these quantities, and also of the gravity acceleration, should be taken into account. As a preliminary analysis, we tried to find possible connections between *VSH* (and corresponding vertical gradient) and some physical quantities measured by ESA’s Swarm satellites. Specifically, here we present a preliminary comparison between the electron temperature recorded by ESA’s Swarm satellites and the vertical gradient of *VSH*. Electron temperature can be considered as a proxy of plasma temperature. We focus on the vertical gradient of *VSH* because the analysis of the section “Deriving the effective vertical scale height from Radio Occultation data” has demonstrated that the topside ionosphere exhibits a very clear linear trend of the scale height and then a constant scale height gradient. Because the scale height gradient is constant for most of the altitudes sounded by COSMIC satellites, it is fair to use the comparison with Swarm satellites measurements collected between about 450 and 520 km of altitude.

Swarm^38^ is a three-satellites constellation launched at the end of 2013 by ESA in a LEO circular near-polar orbit. Two of them, Swarm A and C, are orbiting the Earth side by side at the same altitude of about 460 km, with an inclination of 87.4° and an east–west separation of 1–1.5° in longitude. Swarm B is flying about 60 km higher, with an inclination of 88° on a different orbit. They are all equipped with identical instruments consisting of high-resolution sensors for measurements of both geomagnetic and electric fields, as well as plasma density and temperature.

Here, we consider Level 1b electron temperature *T*_e_ measurements at 2 Hz rate recorded by the Swarm’s Langmuir probes^39^ from the beginning of 2014 to the end of 2019. Swarm’s data are freely accessible at ftp://swarm-diss.eo.esa.int. Detailed information on Swarm’s Langmuir Probes data are provided in Knudsen et al.^40^ and Lomidze et al.^41^.

**Figure 6:**
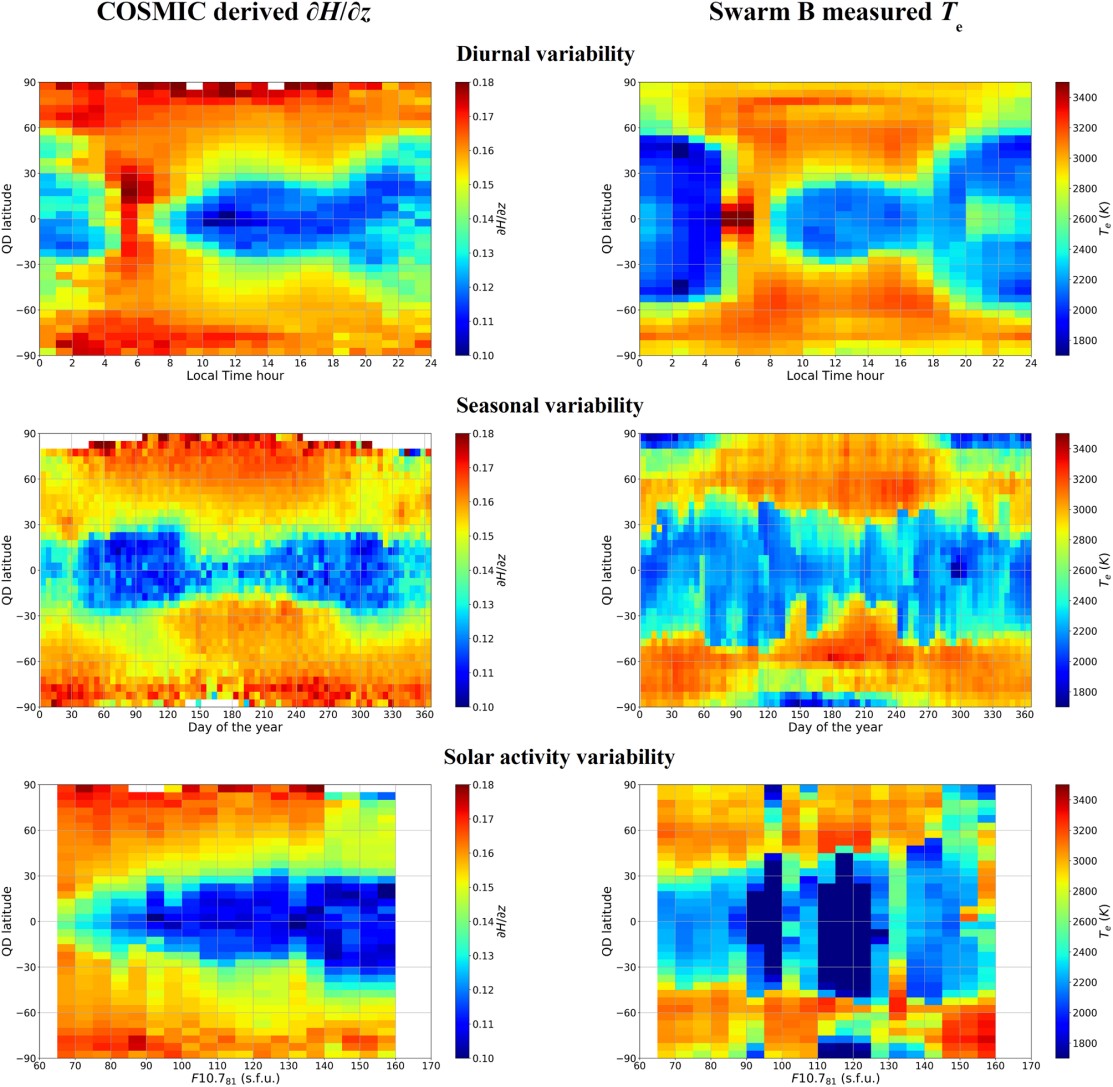
(Left panels) Topside vertical scale height gradient values as deduced from COSMIC RO dataset (2006–2018). Binned median scale height gradient values are shown as a function of QD latitude (*y*-axis) and of (*x*-axis) Local Time hour (top panels), day of the year (middle panels), and F10.7_81_ (bottom panels). (Right panels) Swarm B measured *T*_e_ dataset (2014–2019) binned like the topside vertical scale height gradient values.

Right panels of Fig. 6 show median Swarm B *T*_e_ values binned as a function of Quasi-Dipole^42^ magnetic latitude on y-axis (QD), and of Local Time, day of the year, and 81-days running mean of the solar index F10.7^43^ (F10.7_81_). Left panels of Fig. 6 show instead median values of the calculated topside effective scale height gradient ${\partial{H_{\text{Linear}}}} {\partial{z}}$ derived from COSMIC dataset, as described in the section “Deriving the effective vertical scale height from Radio Occultation data”, and binned as Swarm data. The COSMIC dataset provides a good and quite uniform coverage of different diurnal and seasonal conditions for the QD latitude range ± 70°. In terms of solar activity the dataset is instead slightly biased toward low solar activity values because most of RO profiles were recorded at the beginning of the mission, i.e., for years of low solar activity (2006–2010).

As demonstrated in the sections “Linking the theory to retrieved effective parameters” and “Deriving the effective vertical scale height from Radio Occultation data”, the equalities ${{\partial H_{{{\text{Epstein}}}} } \mathord{\left/ {\vphantom {{\partial H_{{{\text{Epstein}}}} } {\partial z}}} \right. \kern-\nulldelimiterspace} {\partial z}}{{ \equiv \partial H_{{{\text{Linear}}}} } \mathord{\left/ {\vphantom {{ \equiv \partial H_{{{\text{Linear}}}} } {\partial z}}} \right. \kern-\nulldelimiterspace} {\partial z}} \equiv {{\partial VSH} \mathord{\left/ {\vphantom {{\partial VSH} {\partial z}}} \right. \kern-\nulldelimiterspace} {\partial z}}$ can be assumed valid already after a few scale heights above the F2-layer peak. Then, for the sake of brevity, in the following we will refer to ${{\partial H} \mathord{\left/ {\vphantom {{\partial H} {\partial z}}} \right. \kern-\nulldelimiterspace} {\partial z}}$ as the topside vertical scale height gradient. At the altitudes of Swarm B satellite (initial altitude of 520 km) we are usually in a region where a perfect match between effective and theoretical parameters has not yet been achieved (for example, look at lower panels of Figs. 1, 2, and 3 where *n* is between 1.2 and 1.7 at Swarm B altitude). This is why, here we limit ourselves only to the identification of similar climatological patterns between ${{\partial H} \mathord{\left/ {\vphantom {{\partial H} {\partial z}}} \right. \kern-\nulldelimiterspace} {\partial z}}$ and *T*_e_.

Figure 6 shows a remarkable similarity between ${{\partial H} \mathord{\left/ {\vphantom {{\partial H} {\partial z}}} \right. \kern-\nulldelimiterspace} {\partial z}}$ values derived by COSMIC and *T*_e_ measured by Swarm B, when considering the diurnal, seasonal, and solar activity variability for different QD magnetic latitudes. ${{\partial H} \mathord{\left/ {\vphantom {{\partial H} {\partial z}}} \right. \kern-\nulldelimiterspace} {\partial z}}$ and *T*_e_ values exhibit an identical diurnal pattern from equatorial to auroral latitudes. Undoubtedly, the electron temperature (the same holds for plasma temperature) plays a crucial role in driving the diurnal trend of the vertical scale height gradient in the topside ionosphere. Very similar considerations can be made for the seasonal variability. However, in this case *T*_e_ values show a different latitudinal extension of the band of lower values at low latitudes, compared to that of ${{\partial H} \mathord{\left/ {\vphantom {{\partial H} {\partial z}}} \right. \kern-\nulldelimiterspace} {\partial z}}$. Such differences might be attributed to the fact that Swarm B is a near-polar orbit quasi-solar synchronous satellite, which means that the sampling is not evenly distributed. The solar activity plots are the ones showing the worst similarity. This is partly due to the fact that COSMIC and Swarm B datasets cover different years; then, different solar activity levels are unevenly sampled. Both COSMIC and Swarm B datasets are biased toward low solar activity levels, but COSMIC dataset allows for a better coverage of medium and high solar activity levels than Swarm B. Anyway, similar features are shown also in this case with lower values at low latitudes and higher approaching high latitudes, with a tendency to enlarge the latitudes range of low values for high solar activity.

Results of Fig. 6 are only a first attempt to apply what has been discussed about the linking between effective and theoretical parameters and the corresponding comprehension of physical properties of the topside ionosphere. More in depth studies and comparisons with other measured data are needed.

## Conclusions

This paper has shown that it is possible to mathematically link the theoretical vertical scale height *VSH*, that is connected to several physical parameters through the plasma ambipolar diffusion theory, to the effective scale height *H*, as retrieved from the analysis of COSMIC topside electron density profiles through the Epstein formulation. Specifically, the effective scale height *H*_Epstein_(*z*) derived from the semi-Epstein function has been used as the effective parameter because of its fully analytical description. A mathematical relation between *VSH* and *H*_Epstein_(*z*) has then been obtained and corresponding mathematical properties have been studied. It has been demonstrated how *VSH* and *H*_Epstein_(*z*) (and corresponding vertical gradients) tend to each other in the topside ionosphere, from some hundreds of kilometers above the F2-layer peak to the plasmasphere. This means that from the study of *H*_Epstein_(*z*), information can be deduced for *VSH* and then for the physical quantities involved in its variability. Following the approach proposed by Pignalberi et al.^13^, effective scale height values and gradients have been calculated for the entire COSMIC RO dataset. Finally, topside scale height gradient values from the COSMIC dataset have been compared with electron temperature values measured by ESA’s Swarm B satellite. It was found that both exhibit very similar diurnal, seasonal, and solar activity trends as a function of QD magnetic latitude. This study represents only a first attempt to unveil the physical properties of the topside ionosphere starting from calculated effective values. Conversely, the exploitation of different measurement techniques and satellites missions capable of retrieving physical parameters in the topside ionosphere, can help in improving the topside description made by ionospheric models like NeQuick and IRI through the proposed methodology. More in depth studies are needed to fully characterize the effective scale height variability on different helio-geophysical parameters and to link it to the physical quantities involved in the plasma ambipolar diffusion theory.

## Methods

### On the selection and filtering of COSMIC/FORMOSAT-3 RO topside profiles

The initial dataset of 3,626,729 COSMIC ionPrf files, recorded from 22 April 2006 to 31 December 2018, underwent a selection and filtering process constituted by several steps.

Specifically, the initial selection consisted in discarding profiles for which one (or more) of the following conditions is met:

1. *h*_COSMIC_ < *hm*F2 + 150 km; i.e., we require a topside profile at least 150 km wide to make a reliable fit of the topside scale height;
2. *N*_e_ < 0 for at least one point at *h* > *hm*F2;
3. It was not possible to apply the linear fitting procedure of the topside scale height (see Eq. (18)) due to missing or corrupted data in the topside profile;
4. The ionPrf file is corrupted.

After this initial filtering procedure, the original COSMIC dataset reduced to 3,069,418 electron density profiles. Then, 557,311 profiles (about 15.4% of the analyzed dataset) were discarded.

Afterwards, the dataset of 3,069,418 profiles underwent a second filtering procedure where COSMIC profiles were discarded if at least one of the following conditions is met:

- *fo*F2 < 0.1 MHz or *fo*F2 > 22 MHz;
- *hm*F2 < 150 km or *hm*F2 > 650 km;
- $\frac{{\partial H_{{{\text{Linear}}}} }}{\partial z} < 0$. This condition avoids profiles for which the topside *N*_e_ tends to increase for most of the topside profile, which is not physically acceptable;
- $\left| {Lat_{{hm{\text{F2}}}} - Lat_{{hm{\text{F2}} + 150{\text{km}}}} } \right| \ge 5^\circ$ or $\left| {Lon_{{hm{\text{F2}}}} - Lon_{{hm{\text{F2}} + 150{\text{km}}}} } \right| \ge 10^\circ$,where *Lat*_*hm*F2_ and *Lat*_*hm*F2+150 km_ are the geographic latitudes, and *Lon*_*hm*F2_ and *Lon*_*hm*F2+150 km_ the geographic longitudes, of electron density values recorded at *hm*F2 and 150 km above *hm*F2. In this way, too slanted topside profiles were discarded.

After this second filtering procedure, 26,618 (0.87% of 3,069,418) profiles were discarded.

Afterwards, we developed a specific filter to estimate the *noise*, at different spatial scales, of the COSMIC topside electron density profiles. We measured the noise level using the standard deviation of the relative differences between the measured electron density profile and its corresponding smoothed one. The smoothed topside electron density profile is calculated as the running mean of the topside profile with running windows of different length: *small* = 10 km, *medium* = 75 km, and *large* = 150 km.

The filtering algorithm is composed by the following steps:

1. The COSMIC topside profile is preliminary vertically interpolated to obtain an even height resolution of 1 km. In this way, we have *N* = *h*_COSMIC_*-hm*F2 topside electron density measurements, indexed through the index *k* running on the whole topside profile;
2. The smoothed electron density values $\overline{{N_{{{\text{e}}}} }}_{,k}$ are then calculated:
  21 $$\overline{{N_{{{\text{e}}}} }}_{,k} = \frac{1}{2j + 1}\sum\limits_{i = - j}^{j} {N_{{{\text{e}},k + i}} } ,$$
  where $N_{{{\text{e}},k + i}}$ are the values falling inside the window of width 2*j* + 1 centered on the index *k*. The number of *N*_e_ values falling inside the window is 11 (small window), 76 (medium window), and 151 (large window). For the sake of simplicity, the profile smoothed with the small window is called $\overline{{N_{{\text{e}}} }}_{{\text{,small}}}$, the profile smoothed with the medium window $\overline{{N_{{\text{e}}} }}_{{\text{,medium}}}$, and the profile smoothed with the large window $\overline{{N_{{\text{e}}} }}_{{\text{,large}}}$;
3. The relative differences (relative residuals) between measured and smoothed electron density values are calculated:
  22a $$N_{{{\text{e,residuals\_small}}}} = \frac{{N_{{\text{e}}} - \overline{{N_{{\text{e}}} }}_{{\text{,small}}} }}{{\overline{{N_{{\text{e}}} }}_{{\text{,small}}} }} \cdot 100,$$
  22b $$N_{{{\text{e,residuals\_medium}}}} = \frac{{N_{{\text{e}}} - \overline{{N_{{\text{e}}} }}_{{\text{,medium}}} }}{{\overline{{N_{{\text{e}}} }}_{{\text{,medium}}} }} \cdot 100,$$
  22c $$N_{{{\text{e,residuals\_large}}}} = \frac{{N_{{\text{e}}} - \overline{{N_{{\text{e}}} }}_{{\text{,large}}} }}{{\overline{{N_{{\text{e}}} }}_{{\text{,large}}} }} \cdot 100.$$
4. The standard deviation of residuals of Eqs. (22a–c) is calculated, that is what is considered the *noise* in the topside profile:
  23a $${\text{Noise\_small}} = \sqrt {\frac{1}{N - 1}\sum\limits_{i = 1}^{N} {\left( {N_{{{\text{e,residuals\_small}},i}} - \overline{{N_{{\text{e}}} }}_{{{\text{,residuals\_small}}}} } \right)^{2} } } ,$$
  23b $${\text{Noise\_medium}} = \sqrt {\frac{1}{N - 1}\sum\limits_{i = 1}^{N} {\left( {N_{{{\text{e,residuals\_medium}},i}} - \overline{{N_{{\text{e}}} }}_{{{\text{,residuals\_medium}}}} } \right)^{2} } } ,$$
  23c $${\text{Noise\_large}} = \sqrt {\frac{1}{N - 1}\sum\limits_{i = 1}^{N} {\left( {N_{{{\text{e,residuals\_large}},i}} - \overline{{N_{{\text{e}}} }}_{{{\text{,residuals\_large}}}} } \right)^{2} } } ,$$
  where $\overline{{N_{{\text{e}}} }}_{{{\text{,residuals\_small}}}}$ is the mean value of $N_{{{\text{e,residuals\_small}}}}$ calculated over the whole topside profile (the same holds for the medium and large windows);
5. A profile is considered too noisy, and then discarded, when at least one of the three parameters calculated through Eqs. (23a–c) exceeds definite noise threshold values *T*_window_.

After a preliminary testing phase on the Pignalberi et al.^13^ dataset, in which every topside profile was visually checked, we found that a good compromise is to choose the following noise threshold values:

24 $$\left\{ {\begin{array}{*{20}l} {T_{{{\text{small}}}} = 2} \hfill \\ {T_{{{\text{medium}}}} = 3} \hfill \\ {T_{{{\text{large}}}} = 4} \hfill \\ \end{array} } \right..$$

If at least one of these three thresholds is exceeded, the corresponding COSMIC topside profile is discarded.

After applying the above described filtering technique, 1,251,124 (40.76% of 3,069,418) profiles were discarded.

Finally, the COSMIC dataset considered in this work is constituted by 1,791,676 (58.37% of 3,069,418) topside profiles.
